# Analysis of vapor cloud explosion behavior in LPG spherical tanks using multi-energy modeling and machine-learning-based factor assessment

**DOI:** 10.1371/journal.pone.0353909

**Published:** 2026-07-16

**Authors:** Keyvan Sarebanzadeh, Mahboubeh Es’haghi

**Affiliations:** Department of Occupational Health Engineering and Safety at Work, Faculty of Public Health, Kerman University of Medical Sciences, Kerman, Iran; University of Salerno: Universita degli Studi di Salerno, ITALY

## Abstract

Vapor cloud explosions (VCEs) associated with liquefied petroleum gas (LPG) storage systems represent a significant hazard in refinery operations. Accurate estimation of explosion distance is essential for safety setback design and quantitative risk assessment. This study developed an integrated framework that combines physics-based and data-driven techniques to study VCE behavior resulting from LPG releases from a 20,000-barrel pressurized spherical tank under realistic refinery conditions. A total of 336 leakage scenarios were simulated using DNV PHAST 2022 and the Multi-Energy method. Seven influencing parameters were considered, including leak diameter, LPG composition (propane–butane ratio), leak location, atmospheric category, seasonal period, day/night conditions, and three overpressure thresholds (0.02, 0.14, and 0.21 bar). The results revealed a wide dispersion in explosion distance across scenarios, with maximum distances exceeding 2,100 m for catastrophic full-bore ruptures. Feature-importance analysis using a Random Forest regression model showed that leak diameter was the dominant controlling parameter, accounting for approximately 74% of the predictive importance, while LPG composition contributed an additional 24–25%. Operational and atmospheric parameters had comparatively minor effects. The three investigated overpressure thresholds produced nearly identical explosion distances across the evaluated scenarios, indicating that within the low-pressure range, explosion extent is primarily governed by the released fuel mass and flammable cloud geometry rather than the threshold value. A classical power-law correlation was used to describe the geometric scaling between leak diameter and explosion distance; however, its predictive capability was limited (RMSE ≈ 337 m) due to its single-variable formulation. In contrast, the Random Forest model captured multi-factor interactions within the PHAST-generated dataset with very high predictive accuracy (R² ≈ 0.9997, RMSE ≈ 9 m). This hybrid framework provides a transparent approach for analyzing LPG vapor cloud explosions and offers practical insights for safety-distance determination, refinery layout optimization, and risk-based inspection planning.

## 1. Introduction

Vapor cloud explosions (VCEs) present a significant challenge in the oil, gas, and petrochemical industries, particularly in facilities handling liquefied petroleum gases (LPG) such as propane and butane mixtures. Large-scale industrial accidents, such as Flixborough (1974) [1], Texas City (2005) [2], and Buncefield (2005) [3], have shown that accidental LPG releases can lead to the formation of large flammable vapor clouds. When ignited, these clouds can generate destructive overpressure waves capable of affecting areas ranging from hundreds of meters to several kilometers [4–6]. These events pose significant risks to personnel safety, critical infrastructure, and surrounding communities.

Accurate estimation of explosion distances is essential for safety-distance determination, refinery layout design, land-use planning, and quantitative risk assessment (QRA). In practice, consequence modeling tools such as PHAST are widely used to simulate gas dispersion, flammable cloud formation, and explosion overpressure under realistic operating conditions [7–9]. While these tools provide physically grounded and validated predictions [10,11], their outputs are often analyzed using simplified or scenario-based approaches, which may limit the systematic understanding of parameter interactions. In parallel, simplified analytical models, such as power-law correlations between leak diameter and explosion distance, are frequently used for preliminary hazard assessment because of their simplicity. However, these models are inherently limited to single-variable relationships and cannot adequately represent the combined effects of fuel composition, operational conditions, and environmental variability, particularly in complex refinery scenarios.

Recent advancements in data-driven methods have introduced machine-learning (ML) techniques as powerful tools for capturing nonlinear relationships in complex systems. Although ML models have shown high predictive accuracy in process safety applications, their integration with established physics-based consequence models remains limited [12,13]. Most existing studies either focus solely on predictive performance without physical interpretability or fail to explicitly quantify the relative importance of governing parameters. Therefore, a key challenge remains in bridging the gap between validated physics-based modeling and interpretable multi-factor analysis.

Unlike previous studies that focused either on deterministic consequence modeling or purely data-driven prediction, this study integrates validated PHAST-based Multi-Energy simulations with an interpretable Random Forest regression framework to quantify the relative importance of governing parameters affecting VCE distance. The analysis focuses on LPG releases from a pressurized spherical storage tank under realistic refinery operating conditions. The objectives are to (i) quantify the relative influence of leak diameter, fuel composition, and operational conditions on explosion distance, (ii) evaluate the adequacy of classical power-law scaling, and (iii) provide physically interpretable insights to support safety-distance determination and risk-informed decision-making in refinery environments.

## 2. Literature review

Vapor cloud explosions have been extensively studied using experimental, analytical, and numerical approaches because of their significant impact on process industries. Early large-scale experiments and subsequent analytical developments have shown that the severity of these explosions depends on factors such as the mass of fuel involved, the size and geometry of the flammable cloud, and the level of congestion or confinement in the surrounding environment. Foundational studies referenced by [7–9] indicate that turbulence generation and cloud dimensions, rather than ignition energy alone, are the primary factors controlling flame acceleration and overpressure development.

With the advancement of computational power, numerical simulation has become the primary method for detailed VCE analysis. Computational Fluid Dynamics (CFD) software such as FLACS and OpenFOAM enable precise modeling of gas dispersion, ignition, flame propagation, and obstacle interactions in complex industrial settings. Several studies have shown that confined or congested environments can significantly increase overpressure levels, while cloud density and fuel composition are crucial factors in determining explosion intensity [14,15]. Recent CFD-based investigations have further confirmed that flame acceleration and overpressure amplification are strongly influenced by turbulence intensity, obstacle density, and vapor cloud heterogeneity [16–19]. Despite their high accuracy, CFD-based models require detailed geometric information and significant computational resources, which limits their applicability in large-scale parametric or probabilistic risk studies.

For engineering practice and regulatory applications, validated consequence models are essential. PHAST, developed by DNV, is one of the most widely used tools for dispersion and explosion consequence modeling. It incorporates established methodologies such as the Multi-Energy Method and the Baker–Strehlow–Tang (BST) model, both grounded in extensive experimental datasets and adopted in international standards and guidelines [8,20,21]. The Multi-Energy Method is particularly suitable for open or semi-confined environments and provides conservative yet practical estimates of explosion distances. Recent applications demonstrate that PHAST remains a key tool in QRA, refinery siting, and land-use planning [22].

Numerous studies have utilized PHAST to assess the consequences of VCE resulting from LPG and light-hydrocarbon releases in storage tanks, pipelines, and processing units. These studies have shown that PHAST is effective for scenario-based consequence evaluation and safety-distance estimation [7,14,15]. Recent studies also show that integrating PHAST with sensitivity and parametric analyses improves understanding of the influence of leak size, atmospheric conditions, and fuel composition [23–25]. However, PHAST relies on simplified geometrical assumptions and predefined energy levels, and its outputs are typically interpreted deterministically. Consequently, the relative impact of key parameters such as leak size, fuel composition, meteorological conditions, and operational variables is rarely quantified systematically.

To facilitate hazard screening, various empirical and semi-empirical correlations have been proposed to establish relationships between explosion distance and leak size or released mass. Power-law relationships are commonly used because of their simplicity. Previous studies have demonstrated sub-linear scaling between damage distance and release size, indicating increased cloud dilution and dissipative effects at larger scales [8,15]. More recent analyses confirm that while power-law correlations capture first-order geometric trends, their predictive performance decreases under multi-variable conditions involving fuel composition and atmospheric variability [26,27]. However, the single-variable nature of these correlations limits their ability to represent compositional and environmental variations, reducing predictive accuracy for complex or large-scale scenarios.

In recent years, machine-learning techniques have been increasingly applied in process safety engineering for accident prediction, risk classification, and consequence modeling [20,28–30]. Algorithms such as artificial neural networks, support vector machines, and k-nearest neighbors have been used to approximate CFD outputs or classify hazardous scenarios [31–37]. Random Forest, in particular, has gained attention because of its robustness, resistance to overfitting, ability to handle mixed data types, and capability to quantify feature importance in an interpretable manner. Recent studies demonstrate that ML models trained on simulation or historical data can achieve high predictive accuracy (R² > 0.95) for explosion overpressure and hazard-distance estimation [38–40].

Although previous studies have significantly advanced VCE consequence modeling, a systematic framework that integrates validated PHAST simulations with interpretable machine-learning analysis for LPG storage scenarios remains limited. In particular, the relative contribution of leak geometry, fuel composition, and operational parameters to explosion distance has not been quantitatively compared within a unified framework. In addition, most ML-based VCE studies focus primarily on predictive accuracy and often treat the model as a black box, limiting their practical integration into engineering decision-making.

Recent literature highlights the need for hybrid frameworks that combine physics-based modeling with interpretable machine-learning techniques to improve both prediction and physical understanding. However, the systematic coupling of ML-based factor assessment with validated consequence models such as PHAST, particularly for LPG storage scenarios, remains limited in the open literature. This study addresses this gap by combining consequence simulation with feature-importance-based Random Forest modeling to provide both predictive accuracy and physically interpretable decision support.

## 3. Materials and methods

This study aimed to quantify the VCE behavior resulting from accidental releases of LPG from a pressurized spherical storage tank located in an industrial refinery in Iran. The tank has a nominal capacity of 20,000 barrels (3,180 m³) and typically operates at 40°C and 8.2 barg, with a design pressure of 18.8 barg and a pressure safety valve (PSV) set at 19.4 barg. The stored material is refinery-grade LPG, primarily consisting of propane (C₃H₈) and butane (C₄H₁₀). To account for realistic seasonal variations in product quality, three mixtures were used in the simulations: 15/85, 30/70, and 50/50 propane–butane by volume. These compositions are in line with typical commercial supply conditions and follow the specifications reported in AIChE-CCPS guidelines, as well as standard references like Casal (2017) [7] and Lees (2012) [9].

Leak scenarios were developed based on historical refinery failure records, expert consultations, and international references [41–44]. Five circular leak diameters were chosen to represent different failure modes including 5 mm (small operational leakage), 25 mm (medium leak), 50 mm (major leak), 100 mm (large release), and 805 mm (full-bore rupture). The selected leak diameters were defined to systematically represent a wide spectrum of credible failure scenarios. Specifically, small diameters (5 and 25 mm) correspond to typical operational leakage, such as flange leakage, valve failure, or corrosion-induced pinhole leaks. Intermediate diameters (50 and 100 mm) represent pipe rupture or mechanical damage scenarios frequently observed in refinery systems. The largest diameter (805 mm) corresponds to a full-bore rupture of the outlet line, representing a worst-case catastrophic failure scenario. This classification is consistent with CCPS recommendations and previous explosion consequence modeling studies.

Each leak size was modeled at both the top and bottom sections of the spherical tank, representing credible corrosion, mechanical damage, and nozzle failure locations observed in refinery inspections. Three operational variables were also included due to their influence on atmospheric stability and dispersion, including leak position (top/ bottom), time of occurrence (day/ night), and seasonal period (first half/ second half of the year).

Explosion and dispersion simulations were conducted using DNV PHAST 2022, a widely used engineering tool for industrial VCE modeling. The software simulates pressurized release, atmospheric dispersion, and vapor cloud formation, followed by explosion modeling using the Multi-Energy method. In this framework, explosion energy levels are assigned based on turbulence and congestion conditions, and overpressure–distance relationships are calculated using standardized blast curves derived from experimental data.

To provide a quantitative interpretation of explosion severity within the Multi-Energy framework, the explosion energy is expressed as:


$$\begin{array}{c} \text{E}=\eta \;\times \;{\text{M}}_{\text{f}}\;\times \;{\Delta \text{H}}_{\text{c}} \end{array}$$

Where E represents explosion energy, Mf is the flammable mass, ΔHc is the heat of combustion, and η is the explosion efficiency factor. This formulation reflects established VCE theory and highlights the dependence of explosion severity on fuel mass and energy release [7,9,45]. In PHAST, Mf is obtained from dispersion modeling, while η is implicitly represented through Multi-Energy levels derived from standardized blast curves. Larger leak diameters result in higher mass release rates and larger vapor clouds, leading to nonlinear amplification of explosion consequences [46]. This approach ensures that explosion source strength is physically grounded rather than arbitrarily defined.

**Fig 1:**
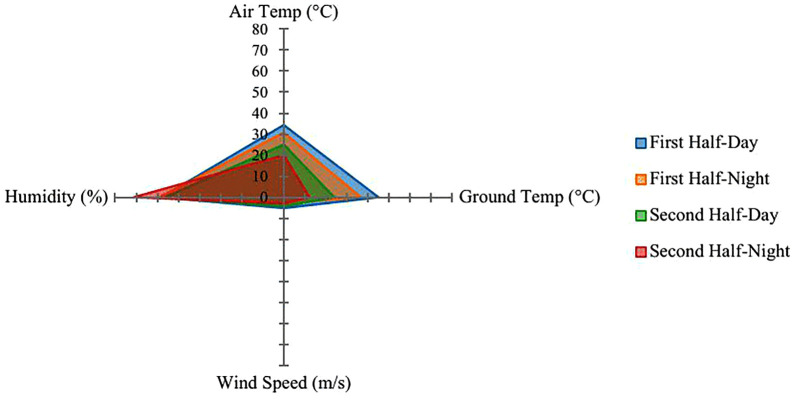
A radar chart comparison of key meteorological parameters.

Three overpressure thresholds were selected to represent different levels of structural damage: 0.02 bar (tolerable structural damage), 0.14 bar (moderate building damage), and 0.21 bar (severe structural failure). Meteorological conditions, including wind speed, ambient temperature, atmospheric stability classes (D, E, F), and humidity, were obtained from refinery climatological records and then integrated into PHAST simulations (Fig 1). PHAST generated overpressure–distance profiles for each combination of leak diameter, LPG composition, overpressure threshold, leak position, time of day, and seasonal period. Some physically non-credible combinations were excluded after PHAST screening. As a result, a total of 336 valid VCE scenarios (112 per overpressure threshold) were simulated, consistent with sample sizes adopted in previous explosion-consequence studies [1,7,17,18,43,47,48].

The results from all PHAST runs were combined into a structured dataset. Any inconsistent, duplicate, or physically non-credible entries were removed during preprocessing. Categorical variables (leak position, day/night, and seasonal period) were encoded using one-hot encoding. The final dataset included input variables (leak diameter, propane fraction, butane fraction, overpressure level, leak position, time of day, and seasonal period) and one response variable: explosion distance (m), consistent with standard practices in VCE consequence modeling.

To characterize the first-order geometric relationship between leak diameter and explosion distance, a classical power-law correlation was applied. This formulation is widely used in blast-wave attenuation and vapor cloud explosion scaling [7,49–51].The general model is expressed as:


$$\begin{array}{c} \text{ln}(\text{L})=\text{ ln}(\text{a})\;+\text{ b ln}(\text{D}) \end{array}$$

Where L is the explosion distance (m), D is the leak diameter (mm), a is the scaling coefficient, and b is the sensitivity exponent. The parameters were estimated using ordinary least squares regression in logarithmic space. The model was fitted using (i) all scenarios and (ii) separately for each overpressure threshold. Since this model is purely geometric, it does not account for compositional or operational variables.

To capture nonlinear interactions among variables, a Random Forest regression model was developed. The input features included leak diameter, propane fraction, butane fraction, overpressure level, leak position, time of day, and seasonal period. The dataset was divided into training (80%) and testing (20%) subsets. The model was implemented with 500 trees, unrestricted maximum tree depth, and the mean-squared-error splitting criterion, following recommended practices for engineering applications [52–54]. The prediction function is expressed as:


$$\begin{array}{c} \hat{y}\;=\;(\text{1}/\text{T})\;\Sigma \;{\text{f}}_{\text{t}}(\text{x}) \end{array}$$

Where $\hat{y}$ is the predicted explosion distance, T is the total number of trees, ${\text{f}}_{\text{t}}(\text{x})$ is the prediction of tree t, and x is the vector of input features. Model performance was evaluated using R², RMSE, and MAE. No significant overfitting was observed, as indicated by the close agreement between training and testing performance. Feature importance analysis was conducted to quantify the contribution of each variable.

**Fig 2:**
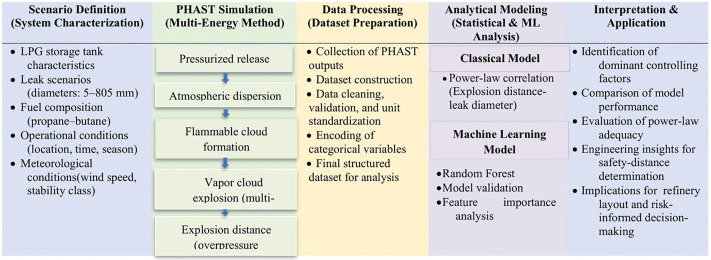
Research workflow including scenario definition, PHAST simulation, data processing, and machine-learning analysis.

For clarity and reproducibility, the overall methodology is summarized in Fig 2. The process includes: (i) system definition and scenario selection, (ii) PHAST-based simulation of release, dispersion, and explosion, (iii) data preprocessing and dataset construction, and (iv) analytical modeling using power-law and Random Forest approaches. This structured workflow provides a clear link between physics-based simulation and data-driven analysis.

Due to the inherent limitations of full-scale VCE experiments, validated numerical approaches are commonly used. PHAST is widely accepted for consequence analysis in QRA and refinery safety studies. Additionally, CFD-based studies show that numerical models can predict explosion overpressure with acceptable accuracy (typically within 10%) [55]. The Random Forest model was validated using independent test data and demonstrated high predictive accuracy with excellent agreement between predicted and simulated explosion distances. Previous studies confirm that ML models can achieve R² > 0.95 in VCE prediction tasks [38]. The agreement between simulation results, machine-learning outputs, and well-established physical trends reported in the literature supports the robustness and reliability of the proposed methodology. All input variables used in the study are summarized in Table 1.

**Table 1:** Summary of model input variables used in PHAST and Random Forest analysis.

Variable	Type	Unit	Role in Model	Description
Leak diameter	Numerical	mm	Primary geometric variable	Controls mass-release rate and initial flammable cloud size
Propane fraction	Numerical	%	Fuel composition variable	Determines vapor density and dispersion rate
Butane fraction	Numerical	%	Fuel composition variable	Affects cloud stability and flame acceleration potential
Overpressure threshold	Numerical/ Categorical	bar	Hazard criterion	Defines damage-distance cutoff (0.02/ 0.14/ 0.21 bar)
Leak position	Categorical	Top/Bottom	Cloud formation setting	Represents credible failure locations in spherical tanks
Time of occurrence	Categorical	Day/Night	Atmospheric condition	Influences stability class and mixing potential
Seasonal period	Categorical	First/Second half	Meteorological condition	Captures seasonal ambient variations relevant to LPG handling
Explosion distance	Numerical	m	Output variable	Hazard radius to be quantified

## 4. Results

### 4.1. Overall statistical behavior of explosion distance

A total of 336 explosion scenarios were simulated for three overpressure thresholds (0.02, 0.14, and 0.21 bar). The descriptive statistics in Table 2 show that explosion distance exhibits an extremely wide dynamic range. Although the mean distance is similar across thresholds (approximately 389–410 m), the standard deviation is very high (approximately 609 m), indicating a strongly right-skewed distribution dominated by a few very large distances. Minimum distances are in the range of 12–33 m, whereas maximum values exceed 2,150 m for all three overpressure levels. This pattern indicates that a small subset of scenarios, corresponding mainly to large leak diameters, produces disproportionately long hazard ranges, while most scenarios remain at much shorter distances. A key observation is that the three overpressure thresholds yield almost identical distance statistics. The mean, median, and upper percentiles differ only slightly between 0.02, 0.14, and 0.21 bar.

**Table 2:** Descriptive statistics of explosion distance for each overpressure level.

Overpressure (bar)	n	Mean Distance (m)	Std. Dev. (m)	Min (m)	25th percentile (m)	Median (m)	75th percentile (m)	Max (m)
0.02	112	409.68	609.45	33.39	53.60	113.76	406.01	2173.45
0.14	112	389.65	609.45	13.54	33.78	93.76	386.09	2153.55
0.21	112	388.29	609.45	12.16	32.23	92.24	384.70	2152.21

### 4.2. Dominant influence of leak diameter on explosion distance

When the data is grouped by leak diameter, a strong and monotonic dependence of explosion distance on orifice size becomes evident (Table 3). For the smallest leaks (5 mm), distances are tightly clustered around 12–34 m with almost zero variance, indicating nearly identical explosion behavior across all operating conditions and overpressure thresholds. This reflects the limited mass release and small reactive cloud associated with micro-leaks. As the leak diameter increases to 25, 50, and 100 mm, explosion distances increase sharply, reaching several hundred meters. Both the mean and upper quartiles (75th percentile) grow with diameter, and variability (standard deviation) becomes progressively larger, reflecting greater sensitivity to atmospheric and compositional conditions. This increasing divergence between small and large leaks is primarily attributed to the nonlinear growth of flammable cloud mass and enhanced turbulence generation at higher release rates, which significantly amplifies explosion overpressure and propagation distance. For catastrophic failure scenarios (805 mm), mean distances exceed 1,360–1,390 m, while maximum distances surpass 2,150 m for all overpressure levels. The standard deviation also rises dramatically (approximately 672 m), indicating a broad spread of possible blast ranges due to the combined effects of increased mass flux, turbulent air entrainment, and expanded cloud geometry.

**Table 3:** Distance statistics by leak diameter for three overpressure levels.

0.02 bar							
**Leak Diameter (mm)**	**Mean (m)**	**Std. Dev. (m)**	**Min (m)**	**25% (m)**	**Median (m)**	**75% (m)**	Max (m)
5	33.62	0.16	33.42	33.50	33.62	33.73	33.85
25	74.03	34.22	33.39	33.75	73.62	106.12	123.85
50	135.69	77.19	53.39	53.75	118.61	218.75	263.45
100	290.27	153.43	83.39	103.58	333.61	418.75	513.45
805	1389.44	671.63	373.40	568.58	1738.54	1876.13	2173.45
**0.14 bar**							
5	13.56	0.03	13.54	13.55	13.56	13.57	13.61
25	54.20	34.21	13.55	13.57	53.79	86.53	105.61
50	115.87	77.19	33.55	33.57	99.31	199.33	245.55
100	270.46	153.43	63.55	83.80	313.81	399.02	498.55
805	1369.63	671.63	353.55	548.83	1718.74	1856.34	2153.55
**0.21 bar**							
5	12.22	0.03	12.16	12.21	12.23	12.23	12.25
25	52.64	34.20	12.20	12.24	52.23	84.73	102.25
50	114.31	77.18	32.20	32.24	97.22	197.24	242.21
100	268.89	153.42	62.20	82.23	312.22	397.24	492.21
805	1368.05	671.63	352.20	547.20	1717.21	1854.72	2152.21

### 4.3. Effect of overpressure threshold

**Fig 3:**
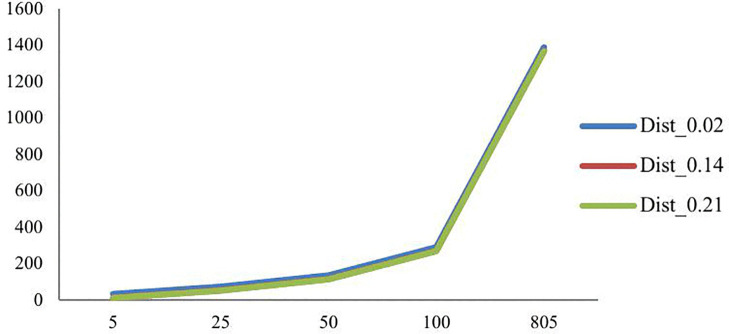
Explosion distance vs leak diameter for 0.02, 0.14, and 0.21 bar.

Despite the conceptual differences between the three overpressure thresholds, the explosion distances at a given diameter are almost identical for 0.02, 0.14, and 0.21 bars. This confirms that, for a given cloud mass and geometry, the overpressure-distance decay is largely insensitive to threshold selection. Fig 3 visualizes this behavior, showing nearly overlapping explosion-distance curves across all leak diameters for the three overpressure thresholds.

**Fig 4:**
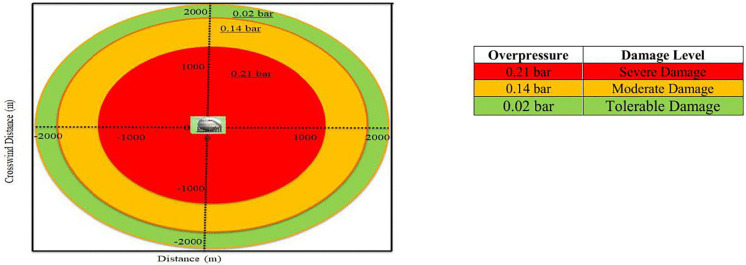
Explosion damage zones for a catastrophic LPG release (805 mm leak diameter) under three overpressure thresholds (0.02, 0.14, and 0.21 bar).

Fig 4 illustrates the VCE damage zones for a catastrophic LPG tank rupture with an 805-mm leak diameter simulated in DNV PHAST 2022. The three concentric circles correspond to overpressure thresholds of 0.02, 0.14, and 0.21 bar, representing tolerable, moderate, and severe damage levels, respectively. The figure visually depicts the spatial extent of the predicted explosion impact zones obtained using the Multi-Energy Method under actual refinery meteorological conditions.

### 4.4. Power-law modeling results

Fitting the overall power-law model to the complete set of 336 scenarios yielded scale and exponent values of a = 3.70 and b = 0.86, with an overall coefficient of determination of R² = 0.69, an RMSE of 337.3 m, and an MAE of 203.3 m. Separate regressions performed for each overpressure threshold showed improved log–log fits, with R² values ranging from 0.795 to 0.802 (Table 4), indicating that threshold-specific fitting better captures the geometric scaling behavior of explosion distance. The lower overall R² compared to threshold-specific models reflects the increased variability introduced when all scenarios are combined across different overpressure conditions. The exponent b < 1 indicates a distinctly sub-linear scaling behavior: although explosion distance increases with leak diameter, the rate of increase diminishes as the diameter grows. This pattern is consistent with dissipative effects of atmospheric turbulence, buoyancy, and air entrainment, which limit the spatial expansion of the blast wave as cloud size increases. The exponents corresponding to 0.02, 0.14, and 0.21 bar were 0.739, 0.904, and 0.923, respectively, demonstrating that all thresholds exhibit sub-linear growth. The upward trend in the exponent values suggests that explosion distance becomes slightly more sensitive to leak diameter under higher overpressure thresholds; however, even at the highest level examined, the growth remained sub-linear. Despite explaining approximately 80% of the variance in logarithmic space for threshold-specific regressions, the relatively large prediction error of the model underscores the limitations of relying on a single geometric variable and points to the necessity of adopting more flexible, multi-factor predictive models for accurate explosion distance estimation (Table 4).

**Table 4:** Power-law relationship between leak diameter and explosion distance.

Overpressure (bar)	n	a (scale)	b (exponent)	R² (log–log)
0.02	112	7.50	0.739	0.795
0.14	112	2.75	0.904	0.802
0.21	112	2.46	0.923	0.802

**Fig 5:**
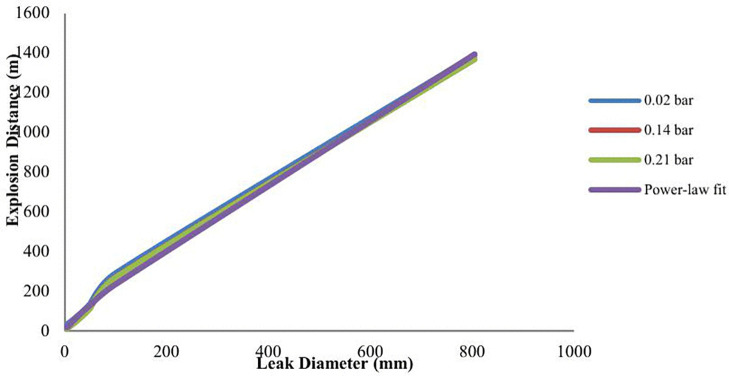
Log-log plot of leak diameter vs explosion distance.

For clarity and improved comparison, all plots use consistent axis scales, units, and axis labels across different overpressure thresholds. Fig 5 illustrates the log–log relationship between leak diameter and explosion distance, along with the fitted power-law curve. This plot highlights two distinct regions corresponding to small (5–25 mm) and large (50–805 mm) leak diameters, emphasizing the transition from weak to strongly nonlinear scaling behavior. For all three overpressure thresholds, the exponent b remains below 1, confirming the sub-linear geometric scaling behavior. The linear trend observed in logarithmic space indicates that explosion distance increases with leak diameter, but at a diminishing rate as expected due to dissipative mechanisms such as turbulence and air entrainment. The slight increase in the exponent b from 0.739 to 0.923 with increasing overpressure suggests that, at higher thresholds, explosion distance becomes marginally more sensitive to leak diameter, consistent with greater blast inertia as larger amounts of fuel contribute to the vapor cloud explosion.

**Fig 6:**
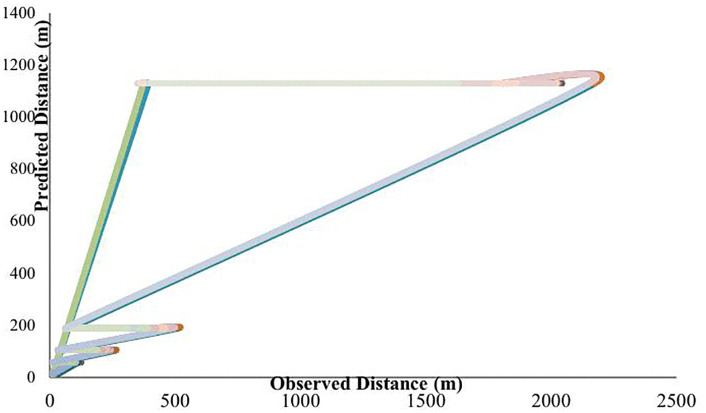
Predicted vs. observed explosion distance using the power-law model.

Fig 6 presents a scatter plot comparing the predicted explosion distances to the observed distances using the classical power-law model. The data points are scattered around the 1:1 reference line, indicating limited predictive accuracy of the model. This limitation is most noticeable for larger leak diameters where deviations are more significant. The deviation from the 1:1 line becomes more pronounced at larger leak diameters, indicating that the power-law model increasingly underperforms in capturing complex multi-factor interactions in high-release scenarios. Although the power-law model captures the general trend of distance increasing with diameter, the dispersion and systematic bias indicate that a single-parameter model cannot fully account for the combined effects of LPG composition, atmospheric dispersion characteristics, and flammable cloud geometry. The root mean square error RMSE of approximately 337 meters further confirms the model’s restricted ability to predict explosion distances under various operating conditions.

### 4.5. Machine-learning model performance

The Random Forest regression model demonstrated excellent predictive performance for explosion distance under varying leak, compositional, and operational conditions. The model achieved an R² of 0.9999 on the training dataset and an R² of 0.9997 on the independent test dataset. The close agreement between training and testing metrics indicates that model overfitting was minimal despite the very high predictive performance. Error values were remarkably low, with RMSE approximately equal to 9.1 m and MAE approximately equal to 5.5 m on the test set. In comparison, the classical power-law correlation had a R² of 0.69 and RMSE of 337.3 m. The Random Forest model reduced prediction error by more than 95%, highlighting its ability to capture complex nonlinear interactions among leak diameter, LPG composition, overpressure level, and operational descriptors. The performance comparison is presented in Table 5.

**Table 5:** Performance comparison between power-law model and Random Forest models.

Model	Data set	R²	RMSE (m)	MAE (m)
Overall Power-law Model	All data	0.69	337.3	203.3
Random Forest	Train	0.9999	6.49	2.74
Random Forest	Test	0.9997	9.13	5.53

**Fig 7:**
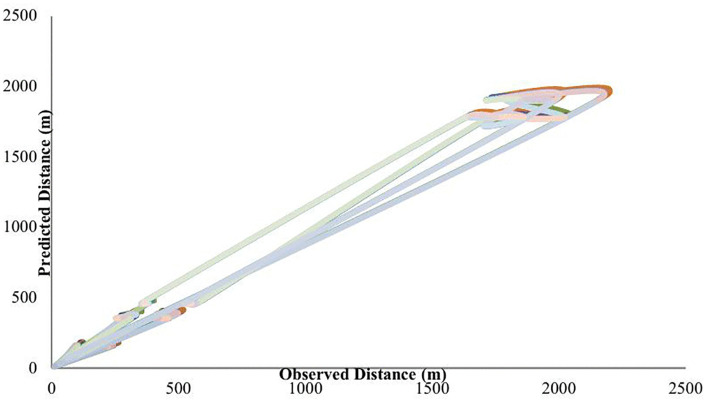
Predicted vs. observed explosion distance using the Random Forest model.

Fig 7 illustrates a scatter plot comparing the predicted and observed explosion distances using the Random Forest regression model. Unlike the classical power-law correlation, the Random Forest shows a remarkably tight clustering of points along the 1:1 reference line, indicating negligible bias and highly accurate predictions across all scenarios. This behavior reflects the model’s ability to capture nonlinear interactions among multiple factors, including leak diameter, LPG composition, overpressure threshold, and operational parameters. The reduction of prediction error by more than 95% relative to the power-law model (RMSE decreasing from ~337 m to ~9 m) confirms that Random Forest provides a far more robust and physically consistent representation of vapor cloud explosion behavior in complex refinery conditions. The improved clustering across the full range of leak diameters, particularly for large-release scenarios, further demonstrates the robustness of the Random Forest model.

### 4.6. Feature importance analysis

A feature importance analysis was conducted to quantify the relative contribution of each input variable to the Random Forest predictions. The ranked importance is reported in Table 6. Leak diameter clearly dominates, accounting for about 74% of the predictive importance, confirming that it is the primary determinant of explosion distance. LPG composition (propane and butane fractions) contributes an additional 24–25%, indicating a secondary but non-negligible influence related to mixture density and cloud persistence. In contrast, leak position, time of day, seasonal half-year, and overpressure level each contributed less than 1% to the model’s explanatory power. This finding supports the conclusion that, under the simulated refinery conditions, geometric and compositional factors govern explosion distance far more strongly than temporal or categorical factors. It also aligns with physical VCE behavior, where the mass and geometry of the flammable cloud control overpressure propagation, while the choice among closely spaced low-overpressure thresholds (0.02–0.21 bar) has only a marginal effect on predicted hazard ranges. Overall, the combined statistical and machine-learning analyses demonstrate that explosion distance in LPG VCE scenarios is primarily governed by leak geometry and fuel composition, with operational and threshold variations playing a secondary role.

**Table 6:** Feature importance in the Random Forest model.

Rank	Feature	Importance (fraction)	Importance (%)
1	Leak_Diameter_mm	0.743	74.3
2	Propane %	0.131	13.1
3	Butane %	0.114	11.4
4	Leak_Position_top	0.0055	0.55
5	Day_Night_Night	0.0052	0.52
6	Half_Year_Second	0.0013	0.13
7	overpressure (bar)	0.0003	0.03

## 5. Discussion

### 5.1. Statistical behavior and dominant role of leak diameter

In this study, the explosion behavior of a gas cloud formed by an LPG leak from a pressurized spherical tank in a refinery was analyzed. The results showed that the explosion distance in all scenarios spans a wide range. Although the mean explosion distances at the three overpressure thresholds of 0.02, 0.14, and 0.21 bar are similar, the standard deviation is large and the data distribution is highly right-skewed. This statistical pattern indicates that only a small number of large-leak scenarios, particularly the 805-mm diameter leak, are responsible for generating very long explosion distances. Smaller leaks, even under various atmospheric stability conditions, mostly fall within short-range distances. This finding is consistent with previous reports from refineries and LPG storage facilities, and highlights that the “total mass of released gas” and the “initial size of the flammable cloud” are the most important factors determining explosion intensity and range [7,56–58].

An examination of the influencing factors showed that the diameter of the leak is the most influential variable, explaining over 70% of the variance in explosion distance. The composition of propane and butane also plays a significant role, accounting for approximately 24% of the model’s influence. This is due to differences in density, dispersion velocity, and the stability of flammable clouds in propane–butane mixtures. In contrast, operational parameters such as leak location, time of occurrence (day/night), and seasonal period (first/second half of the year) had minimal impact, with each contributing less than 1%. This finding is consistent with the thermodynamic and geometric aspects of explosive cloud formation, where the initial cloud mass has a more dominant role than local operational conditions in determining event severity.

### 5.2. Effect of leak diameter and nonlinear scaling behavior

A key finding of this study is the strong nonlinear relationship between leak diameter and explosion distance. While explosion distance increases with leak size, the rate of increase differs significantly between small and large leak scenarios. For small leaks (5–25 mm), explosion distances remain limited and show minimal sensitivity to environmental and operational conditions. This behavior can be attributed to the restricted mass release rate, which leads to the formation of relatively small vapor clouds. In such cases, rapid atmospheric dilution and limited fuel availability prevent significant flame acceleration, resulting in low overpressure generation and short damage distances [7–9]. In contrast, medium to large leaks (50–805 mm) exhibit a markedly different behavior. As the leak diameter increases, the explosion distance grows disproportionately, indicating a nonlinear amplification effect. This phenomenon is primarily governed by the rapid increase in mass release rate, which directly controls the size, density, and persistence of the flammable vapor cloud. Larger releases produce extensive vapor clouds with higher fuel concentration, enabling more efficient flame acceleration and energy release during ignition. This behavior has been widely reported in recent CFD-based and experimental studies, which confirm that turbulence intensity, cloud size, and congestion strongly influence explosion overpressure and propagation [20,51,59–61]. The difference becomes particularly pronounced in the full-bore rupture scenario (805 mm), where the explosion distance reaches up to approximately 1,800–2,150 m. At this scale, the vapor cloud is sufficiently large to sustain turbulent combustion over extended distances, and dissipative mechanisms such as air entrainment, buoyancy, and dilution become less dominant relative to the available chemical energy. As a result, a greater fraction of the released fuel contributes to blast effects; significantly increasing the overpressure propagation range [7,51,59,60]. This transition reflects a shift from dispersion-controlled behavior in small leaks to fuel-controlled combustion in large releases, explaining why the difference in explosion distance becomes increasingly pronounced with leak diameter. It also explains the statistical skewness observed in the results, where a limited number of large-leak scenarios dominate the upper tail of the explosion distance distribution.

### 5.3. Effect of overpressure threshold

Another important finding of this study is that the explosion distance remains nearly the same for the three overpressure thresholds used (0.02, 0.14, and 0.21 bar). According to the PHAST Multi-Energy model, the propagation of the overpressure wave after the formation of the flammable cloud depends more on the fuel mass and the geometric size of the cloud than on the selected overpressure threshold. This finding is consistent with previous studies showing that, within low overpressure ranges (typically below 0.3 bar), the pressure–distance decay curve has low sensitivity to the selected threshold, and the difference in hazard distance is usually less than 5–10% [7,51]. Therefore, the similarity of explosion distances for the three chosen thresholds is expected and aligned with the physical behavior of VCE overpressure waves.

### 5.4. Evaluation of power-law model

The results of this study show that the power-law model, serving as a first-order geometric approximation, can replicate the general pattern of the relationship between leak diameter and explosion distance. However, it has significant limitations in accurately predicting VCE behavior. For all overpressure thresholds, the power-law exponent was found to be b < 1, indicating sub-linear scaling. This means that as the leak diameter increases, the explosion distance also increases, but the rate of increase diminishes as the diameter grows larger. This phenomenon aligns with the physical principles of cloud dispersion and overpressure-wave attenuation. In large leaks, part of the explosion energy is dissipated through turbulence, buoyancy, and air entrainment as the cloud expands. While the power-law model captures the overall trend, its relatively high prediction error (RMSE ≈ 337 m) indicates that this single-variable model cannot fully account for the multi-factor complexity of VCEs in real refinery conditions. Similar limitations have been reported in recent studies on explosion consequence modeling and scaling relationships [8,20,38,62].

### 5.5. Machine learning interpretation and feature importance

To overcome these limitations, a Random Forest model was used to capture nonlinear interactions among geometric, compositional, and operational variables. The machine-learning model demonstrated significantly higher predictive capability than the power-law formulation. Feature-importance analysis revealed that leak diameter is the most influential parameter, explaining approximately 74% of the variability in explosion distance, followed by LPG composition (approximately 24–25%). In contrast, operational and environmental factors such as time of occurrence, seasonal period, and leak position had minimal individual influence (less than 1%). These findings are consistent with recent machine-learning applications in process safety, which show that ML models can effectively capture nonlinear relationships in explosion and dispersion phenomena with high predictive accuracy [40,63,64]. This also improves model interpretability, allowing engineers to prioritize inspection and mitigation strategies based on the most influential parameters.

### 5.6. Engineering implications and limitations

The findings of this study demonstrate that classical single-factor correlations such as the power-law model provide only an approximate representation of VCE behavior. In contrast, integrating PHAST simulations with a data-driven model such as Random Forest enables a more accurate and practical framework for predicting explosion distances in refinery conditions. From an engineering perspective, the dominant influence of leak size highlights the importance of controlling high-rate releases through improved integrity management, reinforcement of critical nozzles, and targeted inspection of corrosion-sensitive locations. The framework presented can assist in determining safety distances, emergency planning, and refinery layout optimization in line with industry guidelines. Although the Random Forest model showed excellent predictive capability, this study is limited by its reliance on PHAST Multi-Energy simulations and simplified LPG compositions. Future research should incorporate real meteorological data, uncertainty analysis, and high-fidelity CFD tools to better represent complex industrial environments. In addition, the present ML model is trained on simulation-generated data rather than full-scale accident observations, which should be considered when generalizing the results to other refinery configurations.

## 6. Conclusion

This study analyzed 336 VCE scenarios resulting from LPG releases from a pressurized 20,000-barrel spherical storage tank. The results demonstrated that the explosion distance is primarily governed by the geometric characteristics of the release. Among all evaluated parameters, leak diameter was identified as the dominant controlling factor, accounting for approximately 74% of the predictive importance in the Random Forest model. The LPG composition (propane–butane ratio) exhibited a secondary but meaningful influence, contributing about 25% to the variability. In contrast, operational descriptors, including leak position, day/night conditions, and seasonal period, showed comparatively minor effects under the simulated conditions.

A key finding was that the three low overpressure thresholds considered (0.02, 0.14, and 0.21 bar) resulted in nearly identical explosion distances. This indicates that, within this pressure range, the hazard radius is primarily controlled by the mass and geometry of the flammable vapor cloud rather than the selected overpressure criterion.

While the classical power-law model captured the general sub-linear relationship between leak diameter and explosion distance, its relatively high prediction error—particularly for large-scale releases—demonstrates the limitations of single-parameter geometric correlations in complex refinery conditions. In contrast, the Random Forest model successfully captured the nonlinear interactions among geometric, compositional, and operational variables, achieving very high predictive accuracy (R² ≈ 0.9997, RMSE ≈ 9 m). The main contribution of this study lies in integrating a validated physics-based consequence model (PHAST) with an interpretable machine-learning framework to quantify the relative importance of governing parameters in VCE behavior. This hybrid approach provides a robust and practical tool for explosion distance estimation.

From an engineering perspective, the results highlight that risk mitigation strategies should prioritize prevention and control of large-scale releases, as leak diameter and resulting cloud size are the dominant drivers of explosion consequences. The proposed framework can support more reliable safety-distance determination, refinery layout optimization, and risk-informed decision-making, ultimately improving explosion prevention and consequence mitigation in oil and gas facilities.
