# Supplementary material for: Analysis of vapor cloud explosion behavior in LPG spherical tanks using multi-energy modeling and machine-learning-based factor assessment
Source: PLoS One. 2026 Jul 16;21(7):e0353909. doi: 10.1371/journal.pone.0353909 (PMC13374909; doi:10.1371/journal.pone.0353909)
Supplement: Attachment — Submitted filename: Response to Reviewers.docx [file pone.0353909.s002.docx]

**Response to Reviewers**

**Manuscript Title:**
Analysis of Vapor Cloud Explosion Behavior in Spherical LPG Tanks Based on Multi-Energy Modeling and Machine Learning Factor Evaluation

We sincerely thank the Editor and the Reviewers for their thorough review and valuable comments on our manuscript. Their feedback has significantly contributed to improving the scientific quality, clarity, and presentation of the paper. All comments have been carefully considered and addressed in the revised manuscript, and responses are provided below.

To facilitate the review process, revisions in the manuscript are highlighted using two colors. Text shown in **green** indicates restructuring or modification of previously existing content, while text shown in **purple** denotes newly added material introduced in response to the reviewers’ comments. These visual markers are provided solely to improve transparency and ease of review.

**Reviewer #1**

| **Comments** | **Response** |
| --- | --- |
| **Comment 1:** On page 3, the statement 'Five circular leak diameters were chosen to represent different failure modes including 5 mm (small operational leakage), 25 mm (medium leak), 50 mm (major leak), 100 mm (large release), and 805 mm (full-bore rupture)' fails to explain the rationale for selecting these five specific circular leak diameters. | The rationale for selecting the five leak diameters has now been clarified in the *Materials and Methods* section (page 3). Specifically, the selected diameters (5, 25, 50, 100, and 805 mm) represent a wide spectrum of credible failure scenarios based on refinery operational experience and literature. Small diameters (5 and 25 mm) correspond to typical operational leaks (e.g., flange leakage and corrosion pinholes), intermediate diameters (50 and 100 mm) represent pipe rupture or mechanical damage, and the largest diameter (805 mm) corresponds to a full-bore rupture scenario. This classification is consistent with CCPS guidelines and previous explosion consequence modeling studies. |
| **Comment 2:** In the simulation conditions section of Chapter 3, the manuscript mentions variables such as vapor cloud explosion components, leak location, seasonal factors, and time conditions, and lists parameters, but it does not provide a further quantitative description of the explosion source strength. | To address this, a quantitative description of explosion energy has been added to the Materials and Methods section. The explosion source strength is now expressed using the well-established formulation (page 4). This addition strengthens the physical basis of the modeling framework.  E = η × Mf × ΔHc |
| **Comment 3:** In the data analysis part of the 'Results' section, the manuscript describes that the trends in explosion distance changes under three overpressure thresholds are not significantly different, but it fails to explain the reasons behind the trends in explosion distance changes for different leak diameters, such as why the differences between small leaks and large leaks are more pronounced. | Additional explanation has been incorporated in both the *Results* and *Discussion* sections (pages 6 and 11). The stronger divergence between small and large leaks is now explained by the nonlinear increase in mass release rate, which governs the size and persistence of the flammable cloud. Larger leak diameters result in higher fuel mass, enhanced turbulence, and more efficient flame acceleration, leading to significantly larger explosion distances. In contrast, small leaks produce limited vapor clouds that dissipate rapidly, resulting in shorter hazard distances. |
| **Comment 4:** For the figures in the 'Results' chapter, such as Fig. 3, it is recommended to directly annotate or use arrows to highlight key parameters based on the research analysis content, and to unify the color scale range, units, and axis labels. This would significantly enhance the information density and intuitiveness of the images. | All figures in the *Results* section have been revised accordingly. Specifically:   - Key parameters have been highlighted using annotations and arrows where appropriate. - Axis labels, units, and scales have been standardized across all figures. - Color scales have been unified to improve consistency and readability.   These changes enhance the clarity and interpretability of the graphical results. |
| **Comment 5:** For the 'Results' and 'Discussion' chapters, it is suggested to structure them in detailed, hierarchical sections centered on the research objectives of the article to improve the layered argumentation of the paper. | The *Results* and *Discussion* sections have been reorganized into clearer hierarchical subsections aligned with the research objectives. This restructuring improves logical flow, readability, and the overall strength of the argumentation. |
| **Comment 6:** Regarding the overall standardization of the article, the manuscript could be further refined. The citation formats are inconsistent, e.g., [9, 17, 18]. It is recommended to review the entire text and standardize them. Some reference formats have issues, such as Ref.[9]. Please carefully check and ensure they comply with the journal requirements. | The entire manuscript has been carefully reviewed, and all citations and references have been standardized according to the journal guidelines (reference section). Inconsistencies such as formatting issues in Ref. [9] and citation styles (e.g., [9, 17, 18]) have been corrected. |

**Reviewer #2**

| **Comment 1:** 1. Most of the references are from a long time ago. It is recommended to cite the latest research conducted in recent years. | Several recent references (2023–2026) have been added to the Introduction, Literature Review, and Discussion sections to better reflect the current state of research in VCE modeling and machine-learning applications in process safety. |
| --- | --- |
| **Comment 2:** Figures 4 to 6 are difficult to read. The author should make some revisions to enable readers to understand the content more clearly. | **Response:** We appreciate this suggestion. Figures 4–6 have been improved to enhance readability:   - Resolution and font sizes have been increased - Axis labels and legends have been clarified - Visual clutter has been reduced - Consistent formatting has been applied   These revisions significantly improve the clarity of the figures.  (Figures 3-7, Pages 4-10). |
