# Supplementary material for: Analysis of vapor cloud explosion behavior in LPG spherical tanks using multi-energy modeling and machine-learning-based factor assessment
Source: PLoS One. 2026 Jul 16;21(7):e0353909. doi: 10.1371/journal.pone.0353909 (PMC13374909; doi:10.1371/journal.pone.0353909)
Supplement: Attachment — Submitted filename: Response to Reviewer.docx [file pone.0353909.s003.docx]

**Response to Reviewer**

**Manuscript Title:**

Analysis of Vapor Cloud Explosion Behavior in LPG Spherical Tanks Using Multi-Energy Modeling and Machine-Learning-Based Factor Assessment

A revised manuscript with tracked changes has been uploaded. All modifications made in response to the reviewer’s comment are clearly indicated in the tracked-changes version of the manuscript.

Figure 4 was revised in response to reviewer comments received during the previous review round and has been further improved in the current revision to enhance clarity and readability.

**Reviewer Comment:**
"Regarding the figures, I noticed one of them has been revised. Could you please explain the reason for this change? Additionally, the figure is still not clear enough for proper understanding. We suggest the author further revise it."

**Response:**
Figure 4 was revised in the previous revision following reviewer feedback to improve visualization of the explosion impact zones. In the current revision, the figure has been further improved by enhancing image quality, increasing readability, and incorporating clearly distinguishable color-coded overpressure zones with direct labels for the corresponding damage thresholds (0.02, 0.14, and 0.21 bar). These modifications were made solely to improve clarity and interpretation of the results. All changes are shown in the tracked-changes manuscript.
