# Supplementary material for: Analysis of vapor cloud explosion behavior in LPG spherical tanks using multi-energy modeling and machine-learning-based factor assessment
Source: PLoS One. 2026 Jul 16;21(7):e0353909. doi: 10.1371/journal.pone.0353909 (PMC13374909; doi:10.1371/journal.pone.0353909)
Supplement: Peer review history

# Peer review: Decision Letter 0

**DOI:** 10.1371/journal.pone.0353909.r001

**Authors:** Gianluca Genovese

17 Mar 2026

PONE-D-26-08717

Analysis of Vapor Cloud Explosion Behavior in LPG Spherical Tanks Using Multi-Energy Modeling and Machine-Learning-Based Factor Assessment

PLOS One

Dear Dr. Es'haghi,

Thank you for submitting your manuscript to PLOS ONE. After careful consideration, we feel that it has merit but does not fully meet PLOS ONE’s publication criteria as it currently stands. Therefore, we invite you to submit a revised version of the manuscript that addresses the points raised during the review process.

Please submit your revised manuscript by May 01 2026 11:59PM. If you will need more time than this to complete your revisions, please reply to this message or contact the journal office at plosone@plos.org. When you're ready to submit your revision, log on to https://www.editorialmanager.com/pone/ and select the 'Submissions Needing Revision' folder to locate your manuscript file.

Please include the following items when submitting your revised manuscript:

- A letter that responds to each point raised by the academic editor and reviewer(s). You should upload this letter as a separate file labeled 'Response to Reviewers'.
- A marked-up copy of your manuscript that highlights changes made to the original version. You should upload this as a separate file labeled 'Revised Manuscript with Track Changes'.
- An unmarked version of your revised paper without tracked changes. You should upload this as a separate file labeled 'Manuscript'.

-->If you would like to make changes to your financial disclosure, please include your updated statement in your cover letter. Guidelines for resubmitting your figure files are available below the reviewer comments at the end of this letter.

If applicable, we recommend that you deposit your laboratory protocols in protocols.io to enhance the reproducibility of your results. Protocols.io assigns your protocol its own identifier (DOI) so that it can be cited independently in the future. For instructions see: https://journals.plos.org/plosone/s/submission-guidelines#loc-laboratory-protocols. Additionally, PLOS ONE offers an option for publishing peer-reviewed Lab Protocol articles, which describe protocols hosted on protocols.io. Read more information on sharing protocols at https://plos.org/protocols?utm_medium=editorial-email&utm_source=authorletters&utm_campaign=protocols.

We look forward to receiving your revised manuscript.

Kind regards,

Gianluca Genovese, Ph.D.

Academic Editor

PLOS One

Journal Requirements:

When submitting your revision, we need you to address these additional requirements.

1. Please ensure that your manuscript meets PLOS ONE's style requirements, including those for file naming. The PLOS ONE style templates can be found at

https://journals.plos.org/plosone/s/file?id=wjVg/PLOSOne_formatting_sample_main_body.pdf and https://journals.plos.org/plosone/s/file?id=ba62/PLOSOne_formatting_sample_title_authors_affiliations.pdf

2. Please note that PLOS One has specific guidelines on code sharing for submissions in which author-generated code underpins the findings in the manuscript. In these cases, we expect all author-generated code to be made available without restrictions upon publication of the work. Please review our guidelines at https://journals.plos.org/plosone/s/materials-and-software-sharing#loc-sharing-code and ensure that your code is shared in a way that follows best practice and facilitates reproducibility and reuse.

3. Thank you for stating the following in your Competing Interests section:

[NO authors have competing interests].

Please complete your Competing Interests on the online submission form to state any Competing Interests. If you have no competing interests, please state "The authors have declared that no competing interests exist.", as detailed online in our guide for authors at http://journals.plos.org/plosone/s/submit-now

This information should be included in your cover letter; we will change the online submission form on your behalf.

If the reviewer comments include a recommendation to cite specific previously published works, please review and evaluate these publications to determine whether they are relevant and should be cited. There is no requirement to cite these works unless the editor has indicated otherwise.

Please review your reference list to ensure that it is complete and correct. If you have cited papers that have been retracted, please include the rationale for doing so in the manuscript text, or remove these references and replace them with relevant current references. Any changes to the reference list should be mentioned in the rebuttal letter that accompanies your revised manuscript. If you need to cite a retracted article, indicate the article’s retracted status in the References list and also include a citation and full reference for the retraction notice.

[Note: HTML markup is below. Please do not edit.]

Reviewers' comments:

Reviewer's Responses to Questions

**Comments to the Author**

1. Is the manuscript technically sound, and do the data support the conclusions?

The manuscript must describe a technically sound piece of scientific research with data that supports the conclusions. Experiments must have been conducted rigorously, with appropriate controls, replication, and sample sizes. The conclusions must be drawn appropriately based on the data presented.

Reviewer #1: Yes

Reviewer #2: Yes

**********

2. Has the statistical analysis been performed appropriately and rigorously?

Reviewer #1: Yes

Reviewer #2: Yes

**********

3. Have the authors made all data underlying the findings in their manuscript fully available?

The PLOS Data policy requires authors to make all data underlying the findings described in their manuscript fully available without restriction, with rare exception (please refer to the Data Availability Statement in the manuscript PDF file). The data should be provided as part of the manuscript or its supporting information, or deposited to a public repository. For example, in addition to summary statistics, the data points behind means, medians and variance measures should be available. If there are restrictions on publicly sharing data—e.g. participant privacy or use of data from a third party—those must be specified.

Reviewer #1: Yes

Reviewer #2: Yes

**********

4. Is the manuscript presented in an intelligible fashion and written in standard English?

PLOS ONE does not copyedit accepted manuscripts, so the language in submitted articles must be clear, correct, and unambiguous. Any typographical or grammatical errors should be corrected at revision, so please note any specific errors here.

Reviewer #1: Yes

Reviewer #2: Yes

**********

5. Review Comments to the Author

Please use the space provided to explain your answers to the questions above. You may also include additional comments for the author, including concerns about dual publication, research ethics, or publication ethics. (Please upload your review as an attachment if it exceeds 20,000 characters)

Reviewer #1: The manuscript titled 'Analysis of Vapor Cloud Explosion Behavior in Spherical LPG Tanks Based on Multi-Energy Modeling and Machine Learning Factor Evaluation' utilized PHAST multi-energy modeling to simulate 336 vapor cloud explosion scenarios occurring in spherical LPG tanks. Employing Python and a random forest regression model for machine learning modeling and statistical analysis, it systematically examined the explosion distances of pressurized spherical LPG tanks. It quantified the relative impacts of factors such as leak diameter, LPG composition, leak location, atmospheric category, seasonal period, day-night conditions, and three overpressure thresholds on explosion distance, providing reference significance for determining safe distances, optimizing refinery layout, and planning risk-based safety inspections. The analytical approach adopted in the manuscript integrates simulation data with machine learning. Compared to traditional simulation analysis models, it offers a deeper analysis of the interactions among various influencing factors, enhances prediction accuracy, and demonstrates distinct advantages. Overall, the paper is well-structured, clearly expressed, and easy to comprehend; thus, this manuscript can be accepted after minor revisions.

1. On page 3, the statement 'Five circular leak diameters were chosen to represent different failure modes including 5 mm (small operational leakage), 25 mm (medium leak), 50 mm (major leak), 100 mm (large release), and 805 mm (full-bore rupture)' fails to explain the rationale for selecting these five specific circular leak diameters.

2. In the simulation conditions section of Chapter 3, the manuscript mentions variables such as vapor cloud explosion components, leak location, seasonal factors, and time conditions, and lists parameters, but it does not provide a further quantitative description of the explosion source strength.

3. In the data analysis part of the 'Results' section, the manuscript describes that the trends in explosion distance changes under three overpressure thresholds are not significantly different, but it fails to explain the reasons behind the trends in explosion distance changes for different leak diameters, such as why the differences between small leaks and large leaks are more pronounced.

4. For the figures in the 'Results' chapter, such as Fig. 3, it is recommended to directly annotate or use arrows to highlight key parameters based on the research analysis content, and to unify the color scale range, units, and axis labels. This would significantly enhance the information density and intuitiveness of the images.

5. For the 'Results' and 'Discussion' chapters, it is suggested to structure them in detailed, hierarchical sections centered around the research objectives of the article to improve the layered argumentation of the paper.

6. Regarding the overall standardization of the article, the manuscript could be further refined. The citation formats are inconsistent, e.g., [9, 17, 18]. It is recommended to review the entire text and standardize them. Some reference formats have issues, such as Ref.[9]. Please carefully check and ensure they comply with the journal requirements.

Reviewer #2: This paper establishes a hybrid framework as a method for analyzing the consequences of liquefied petroleum gas vapor cloud explosions, which is used to determine safe distances, optimize the layout of refineries, and develop risk-based inspection plans. The article is well-argued, but there are some issues. It is recommended to make revisions and then consider for publication.

1.Most of the references are from a long time ago. It is recommended to cite the latest research conducted in recent years.

2. Figures 4 to 6 are difficult to read. The author should make some revisions to enable readers to understand the content more clearly.

**********

6. PLOS authors have the option to publish the peer review history of their article (what does this mean?). If published, this will include your full peer review and any attached files.

If you choose “no”, your identity will remain anonymous but your review may still be made public.

**Do you want your identity to be public for this peer review?**  For information about this choice, including consent withdrawal, please see our Privacy Policy.

Reviewer #1: No

Reviewer #2: No

**********

[NOTE: If reviewer comments were submitted as an attachment file, they will be attached to this email and accessible via the submission site. Please log into your account, locate the manuscript record, and check for the action link "View Attachments". If this link does not appear, there are no attachment files.]

To ensure your figures meet our technical requirements, please review our figure guidelines: https://journals.plos.org/plosone/s/figures

You may also use PLOS’s free figure tool, NAAS, to help you prepare publication quality figures: https://journals.plos.org/plosone/s/figures#loc-tools-for-figure-preparation.

NAAS will assess whether your figures meet our technical requirements by comparing each figure against our figure specifications.

# Author comment: Author response to Decision Letter 1

**DOI:** 10.1371/journal.pone.0353909.r002

7 May 2026

Response to Reviewers

Manuscript Title:

Analysis of Vapor Cloud Explosion Behavior in Spherical LPG Tanks Based on Multi-Energy Modeling and Machine Learning Factor Evaluation

We sincerely thank the Editor and the Reviewers for their thorough review and valuable comments on our manuscript. Their feedback has significantly contributed to improving the scientific quality, clarity, and presentation of the paper. All comments have been carefully considered and addressed in the revised manuscript, and responses are provided below.

To facilitate the review process, revisions in the manuscript are highlighted using two colors. Text shown in green indicates restructuring or modification of previously existing content, while text shown in purple denotes newly added material introduced in response to the reviewers’ comments. These visual markers are provided solely to improve transparency and ease of review.

Reviewer #1

Comments Response

Comment 1: On page 3, the statement 'Five circular leak diameters were chosen to represent different failure modes including 5 mm (small operational leakage), 25 mm (medium leak), 50 mm (major leak), 100 mm (large release), and 805 mm (full-bore rupture)' fails to explain the rationale for selecting these five specific circular leak diameters.

The rationale for selecting the five leak diameters has now been clarified in the Materials and Methods section (page 3). Specifically, the selected diameters (5, 25, 50, 100, and 805 mm) represent a wide spectrum of credible failure scenarios based on refinery operational experience and literature. Small diameters (5 and 25 mm) correspond to typical operational leaks (e.g., flange leakage and corrosion pinholes), intermediate diameters (50 and 100 mm) represent pipe rupture or mechanical damage, and the largest diameter (805 mm) corresponds to a full-bore rupture scenario. This classification is consistent with CCPS guidelines and previous explosion consequence modeling studies.

Comment 2: In the simulation conditions section of Chapter 3, the manuscript mentions variables such as vapor cloud explosion components, leak location, seasonal factors, and time conditions, and lists parameters, but it does not provide a further quantitative description of the explosion source strength.

To address this, a quantitative description of explosion energy has been added to the Materials and Methods section. The explosion source strength is now expressed using the well-established formulation (page 4). This addition strengthens the physical basis of the modeling framework.

E = η × Mf × ΔHc

Comment 3: In the data analysis part of the 'Results' section, the manuscript describes that the trends in explosion distance changes under three overpressure thresholds are not significantly different, but it fails to explain the reasons behind the trends in explosion distance changes for different leak diameters, such as why the differences between small leaks and large leaks are more pronounced.

Additional explanation has been incorporated in both the Results and Discussion sections (pages 6 and 11). The stronger divergence between small and large leaks is now explained by the nonlinear increase in mass release rate, which governs the size and persistence of the flammable cloud. Larger leak diameters result in higher fuel mass, enhanced turbulence, and more efficient flame acceleration, leading to significantly larger explosion distances. In contrast, small leaks produce limited vapor clouds that dissipate rapidly, resulting in shorter hazard distances.

Comment 4: For the figures in the 'Results' chapter, such as Fig. 3, it is recommended to directly annotate or use arrows to highlight key parameters based on the research analysis content, and to unify the color scale range, units, and axis labels. This would significantly enhance the information density and intuitiveness of the images.

All figures in the Results section have been revised accordingly. Specifically:

• Key parameters have been highlighted using annotations and arrows where appropriate.

• Axis labels, units, and scales have been standardized across all figures.

• Color scales have been unified to improve consistency and readability.

These changes enhance the clarity and interpretability of the graphical results.

Comment 5: For the 'Results' and 'Discussion' chapters, it is suggested to structure them in detailed, hierarchical sections centered on the research objectives of the article to improve the layered argumentation of the paper. The Results and Discussion sections have been reorganized into clearer hierarchical subsections aligned with the research objectives. This restructuring improves logical flow, readability, and the overall strength of the argumentation.

Comment 6: Regarding the overall standardization of the article, the manuscript could be further refined. The citation formats are inconsistent, e.g., [9, 17, 18]. It is recommended to review the entire text and standardize them. Some reference formats have issues, such as Ref.[9]. Please carefully check and ensure they comply with the journal requirements.

The entire manuscript has been carefully reviewed, and all citations and references have been standardized according to the journal guidelines (reference section). Inconsistencies such as formatting issues in Ref. [9] and citation styles (e.g., [9, 17, 18]) have been corrected.

Reviewer #2

Comment 1: 1. Most of the references are from a long time ago. It is recommended to cite the latest research conducted in recent years. Several recent references (2023–2026) have been added to the Introduction, Literature Review, and Discussion sections to better reflect the current state of research in VCE modeling and machine-learning applications in process safety.

Comment 2: Figures 4 to 6 are difficult to read. The author should make some revisions to enable readers to understand the content more clearly.

Response:

We appreciate this suggestion. Figures 4–6 have been improved to enhance readability:

• Resolution and font sizes have been increased

• Axis labels and legends have been clarified

• Visual clutter has been reduced

• Consistent formatting has been applied

These revisions significantly improve the clarity of the figures.

(Figures 3-7, Pages 4-10).

Attachment Submitted filename: Response to Reviewers.docx

# Peer review: Decision Letter 1

**DOI:** 10.1371/journal.pone.0353909.r003

**Authors:** Gianluca Genovese, Gianluca Genovese

28 May 2026

PONE-D-26-08717R1

Analysis of Vapor Cloud Explosion Behavior in LPG Spherical Tanks Using Multi-Energy Modeling and Machine-Learning-Based Factor Assessment

PLOS One

Dear Dr. Es'haghi,

Thank you for submitting your manuscript to PLOS ONE. After careful consideration, we feel that it has merit but does not fully meet PLOS ONE’s publication criteria as it currently stands. Therefore, we invite you to submit a revised version of the manuscript that addresses the points raised during the review process.

Please submit your revised manuscript by Jul 12 2026 11:59PM. If you will need more time than this to complete your revisions, please reply to this message or contact the journal office at plosone@plos.org. When you're ready to submit your revision, log on to https://www.editorialmanager.com/pone/ and select the 'Submissions Needing Revision' folder to locate your manuscript file.

Please include the following items when submitting your revised manuscript:

- A letter that responds to each point raised by the academic editor and reviewer(s). You should upload this letter as a separate file labeled 'Response to Reviewers'.
- A marked-up copy of your manuscript that highlights changes made to the original version. You should upload this as a separate file labeled 'Revised Manuscript with Track Changes'.
- An unmarked version of your revised paper without tracked changes. You should upload this as a separate file labeled 'Manuscript'.

If you would like to make changes to your financial disclosure, please include your updated statement in your cover letter. Guidelines for resubmitting your figure files are available below the reviewer comments at the end of this letter.

If applicable, we recommend that you deposit your laboratory protocols in protocols.io to enhance the reproducibility of your results. Protocols.io assigns your protocol its own identifier (DOI) so that it can be cited independently in the future. For instructions see: https://journals.plos.org/plosone/s/submission-guidelines#loc-laboratory-protocols. Additionally, PLOS ONE offers an option for publishing peer-reviewed Lab Protocol articles, which describe protocols hosted on protocols.io. Read more information on sharing protocols at https://plos.org/protocols?utm_medium=editorial-email&utm_source=authorletters&utm_campaign=protocols.

As the corresponding author, your ORCID iD is verified in the submission system and will appear in the published article. PLOS supports the use of ORCID, and we encourage all coauthors to register for an ORCID iD and use it as well. Please encourage your coauthors to verify their ORCID iD within the submission system before final acceptance, as unverified ORCID iDs will not appear in the published article. *Only*  the individual author can complete the verification step; PLOS staff *cannot*  verify ORCID iDs on behalf of authors.

We look forward to receiving your revised manuscript.

Kind regards,

Gianluca Genovese, Ph.D.

Academic Editor

PLOS One

Journal Requirements:

If the reviewer comments include a recommendation to cite specific previously published works, please review and evaluate these publications to determine whether they are relevant and should be cited. There is no requirement to cite these works unless the editor has indicated otherwise.

Please review your reference list to ensure that it is complete and correct. If you have cited papers that have been retracted, please include the rationale for doing so in the manuscript text, or remove these references and replace them with relevant current references. Any changes to the reference list should be mentioned in the rebuttal letter that accompanies your revised manuscript. If you need to cite a retracted article, indicate the article’s retracted status in the References list and also include a citation and full reference for the retraction notice.

Reviewers' comments:

Reviewer's Responses to Questions

**Comments to the Author**

1. If the authors have adequately addressed your comments raised in a previous round of review and you feel that this manuscript is now acceptable for publication, you may indicate that here to bypass the “Comments to the Author” section, enter your conflict of interest statement in the “Confidential to Editor” section, and submit your "Accept" recommendation.

Reviewer #2: All comments have been addressed

**********

2. Is the manuscript technically sound, and do the data support the conclusions?

The manuscript must describe a technically sound piece of scientific research with data that supports the conclusions. Experiments must have been conducted rigorously, with appropriate controls, replication, and sample sizes. The conclusions must be drawn appropriately based on the data presented.

Reviewer #2: Yes

**********

3. Has the statistical analysis been performed appropriately and rigorously?

Reviewer #2: Yes

**********

4. Have the authors made all data underlying the findings in their manuscript fully available?

The PLOS Data policy requires authors to make all data underlying the findings described in their manuscript fully available without restriction, with rare exception (please refer to the Data Availability Statement in the manuscript PDF file). The data should be provided as part of the manuscript or its supporting information, or deposited to a public repository. For example, in addition to summary statistics, the data points behind means, medians and variance measures should be available. If there are restrictions on publicly sharing data—e.g. participant privacy or use of data from a third party—those must be specified.

Reviewer #2: Yes

**********

5. Is the manuscript presented in an intelligible fashion and written in standard English?

PLOS ONE does not copyedit accepted manuscripts, so the language in submitted articles must be clear, correct, and unambiguous. Any typographical or grammatical errors should be corrected at revision, so please note any specific errors here.

Reviewer #2: Yes

**********

6. Review Comments to the Author

Please use the space provided to explain your answers to the questions above. You may also include additional comments for the author, including concerns about dual publication, research ethics, or publication ethics. (Please upload your review as an attachment if it exceeds 20,000 characters)

Reviewer #2: The author has responded to my review and recommends acceptance. Regarding the figures, I noticed one of them has been revised. Could you please explain the reason for this change? Additionally, the figure is still not clear enough for proper understanding. We suggest the author further revise it.

**********

7. PLOS authors have the option to publish the peer review history of their article (what does this mean?). If published, this will include your full peer review and any attached files.

If you choose “no”, your identity will remain anonymous but your review may still be made public.

**Do you want your identity to be public for this peer review?**  For information about this choice, including consent withdrawal, please see our Privacy Policy.

Reviewer #2: No

**********

[NOTE: If reviewer comments were submitted as an attachment file, they will be attached to this email and accessible via the submission site. Please log into your account, locate the manuscript record, and check for the action link "View Attachments". If this link does not appear, there are no attachment files.]

To ensure your figures meet our technical requirements, please review our figure guidelines: https://journals.plos.org/plosone/s/figures

You may also use PLOS’s free figure tool, NAAS, to help you prepare publication quality figures: https://journals.plos.org/plosone/s/figures#loc-tools-for-figure-preparation.

NAAS will assess whether your figures meet our technical requirements by comparing each figure against our figure specifications.

# Author comment: Author response to Decision Letter 2

**DOI:** 10.1371/journal.pone.0353909.r004

4 Jun 2026

Response to Reviewer

Manuscript Title:

Analysis of Vapor Cloud Explosion Behavior in LPG Spherical Tanks Using Multi-Energy Modeling and Machine-Learning-Based Factor Assessment

We sincerely thank the Editor and the Reviewers for their thorough review and valuable comments on our manuscript. In response to the comment regarding Figure 4, we have further revised the figure to improve its clarity and interpretability.

Attachment Submitted filename: Response to Reviewer.docx

# Editor report: Decision Letter 2

**DOI:** 10.1371/journal.pone.0353909.r005

**Authors:** Gianluca Genovese, Gianluca Genovese, Gianluca Genovese

1 Jul 2026

Analysis of Vapor Cloud Explosion Behavior in LPG Spherical Tanks Using Multi-Energy Modeling and Machine-Learning-Based Factor Assessment

PONE-D-26-08717R2

Dear Dr. Es'haghi,

We’re pleased to inform you that your manuscript has been judged scientifically suitable for publication and will be formally accepted for publication once it meets all outstanding technical requirements.

Within one week, you’ll receive an e-mail detailing the required amendments. When these have been addressed, you’ll receive a formal acceptance letter and your manuscript will be scheduled for publication.

An invoice will be generated when your article is formally accepted. Please note, if your institution has a publishing partnership with PLOS and your article meets the relevant criteria, all or part of your publication costs will be covered. Please make sure your user information is up-to-date by logging into Editorial Manager at Editorial Manager® and clicking the ‘Update My Information' link at the top of the page. For questions related to billing, please contact billing support.

If your institution or institutions have a press office, please notify them about your upcoming paper to help maximize its impact. If they’ll be preparing press materials, please inform our press team as soon as possible -- no later than 48 hours after receiving the formal acceptance. Your manuscript will remain under strict press embargo until 2 pm Eastern Time on the date of publication. For more information, please contact onepress@plos.org.

Kind regards,

Gianluca Genovese, Ph.D.

Academic Editor

PLOS One

# Editor report: Acceptance letter

**DOI:** 10.1371/journal.pone.0353909.r006

**Authors:** Gianluca Genovese, Gianluca Genovese, Gianluca Genovese

PONE-D-26-08717R2

PLOS One

Dear Dr. Es'haghi,

I'm pleased to inform you that your manuscript has been deemed suitable for publication in PLOS One. Congratulations! Your manuscript is now being handed over to our production team.

At this stage, our production department will prepare your paper for publication. This includes ensuring the following:

* All references, tables, and figures are properly cited

* All relevant supporting information is included in the manuscript submission,

* There are no issues that prevent the paper from being properly typeset

You will receive further instructions from the production team, including instructions on how to review your proof when it is ready. Please keep in mind that we are working through a large volume of accepted articles, so please give us a few days to review your paper and let you know the next and final steps.

Lastly, if your institution or institutions have a press office, please let them know about your upcoming paper now to help maximize its impact. If they'll be preparing press materials, please inform our press team within the next 48 hours. Your manuscript will remain under strict press embargo until 2 pm Eastern Time on the date of publication. For more information, please contact onepress@plos.org.

You will receive an invoice from PLOS for your publication fee after your manuscript has reached the completed accept phase. If you receive an email requesting payment before acceptance or for any other service, this may be a phishing scheme. Learn how to identify phishing emails and protect your accounts at https://explore.plos.org/phishing.

If we can help with anything else, please email us at customercare@plos.org.

Thank you for submitting your work to PLOS ONE and supporting open access.

Kind regards,

PLOS ONE Editorial Office Staff

on behalf of

Dr. Gianluca Genovese

Academic Editor

PLOS One
